# Supplementary material for: Exploring the colloid-to-polymer transition for ultra-low crosslinked microgels from three to two dimensions
Source: Nat Commun. 2019 Mar 29;10:1418. doi: 10.1038/s41467-019-09227-5 (PMC6441029; doi:10.1038/s41467-019-09227-5)
Supplement: Supplementary file 1 — Supplementary Information [file 41467_2019_9227_MOESM1_ESM.pdf]

Supplementary Information: Exploring the colloid-to-polymer transition for ultra-low crosslinked microgels from three to two dimensions

W. Richtering et al.*

Supplementary Note I: Labeling of the samples.

The regular 5 mol% crosslinked microgels correspond to the sample *SB-104c*. The ultra-low crosslinked microgels correspond to the sample *MB-ULC-140-PNIPAM*. The linear polymer corresponds to the sample *AG-linear*.

Supplementary Note II: Dynamic Light Scattering.

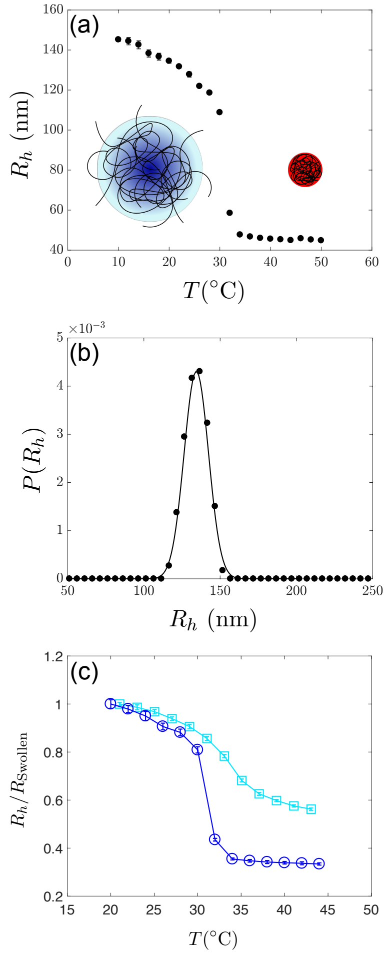

Supplementary Figure 1. DLS data analysis. (a) DLS data of ULC microgels. R_h versus temperature showing the volume phase transition temperature expected for pNIPAM polymers. (b) Contin analysis of DLS data at 20 °C. (c) Swelling ratio, defined as the ratio between the hydrodynamic radius R_h and the hydrodynamic radius at (20.0 ± 0.1) °C, R_{Swollen}, versus the temperature for ultra-low crosslinked and regular 5 mol% crosslinked pNIPAM microgels, ○ and □, respectively.

For the intensity autocorrelation functions, the average decay rate, Γ, was obtained with second cumulant analysis [1]. The decay rates depend on the scattering vector *via* the average diffusion coefficient D_0: $\Gamma = D_0 q^2$. The average diffusion coefficient was obtained by plotting Γ versus q^2 and fitting the data with linear regression [2]. Finally, the Stokes-Einstein relation was used to obtain the hydrodynamic radius R_h: $R_h = k_B T/(6\pi\eta D_0)$ with k_B and η the Boltzmann constant and the viscosity of water at 20 °C, respectively. The values are shown in the second column of Supplementary Table I.

Contin analysis [3, 4] implemented with L-curve criteria [5] has been performed to directly obtain the size distribution of the suspensions and evaluate the size polydispersity, p. The values are shown in the third column of Supplementary Table I.

The swelling behavior of the microgels has been determined measuring the samples between 20 and 50 °C. To compare microgels with different sizes, the ratio between the hydrodynamic radius, R_h, and the radius at 20 °C, R_{Swollen} is plotted in Supplementary Figure 1 for the ultra-low crosslinked and for the 5 mol% crosslinked pNIPAM microgels, ○ and □, respectively. For both microgels, the volume phase transition temperature (VPTT) is at 32 °C. The collapsed size of the regular 5 mol% crosslinked pNIPAM is about 55 % of the swollen radius (153.2 ± 0.6) nm. In contrast, the ULC microgels deswell to (44.8 ± 0.2) nm; ≈ 34 % of their initial size, (134 ± 1) nm.

Supplementary Note III: Swelling ratio and crosslinking.

It is very difficult to estimate the amount of crosslinks incorporated within microgels. It is also impossible to distinguish between crosslinks due to self-crosslinking of pNIPAM promoted by KPS and crosslinks induced by the BIS. However, the swelling behavior below and above the VPTT can be a measure of the softness of the polymeric network. Recently, Lopez and Richtering have collected literature data and found a strong correlation between the effective amount of crosslinker incorporated within a microgel, f, and the hydrodynamic radii below and above the VPTT [6]. The ULC microgels used here were characterized using multi-angle dynamic light scattering to obtain their hydrodynamic radius, R_h, below and above the volume phase transition. We obtained $R_h = (134 \pm 1)$ nm at 20 °C and $R_h = (44.8 \pm 0.2)$ nm at 40 °C. Those values can be used in the relation

Supplementary Table I. Radii and size polydispersities for the samples as obtained from DLS (second and third column) and SANS data (forth and fifth column). Also the conversion constants, k, from viscosimetry are shown. All the values refer to diluted samples at $(20.0 \pm 0.1)\,^{\circ}$C.

**Sample**	**DLS**		**SANS**		**Viscosimetry**
	R_h (nm)	p (%)	R_{SANS} (nm)	σ_{P} (%)	k
ULC	134±1	10 ± 1	126±1	11.3±0.7	44.7±0.1
5 mol% pNIPAM	153.2 ± 0.6	9 ± 3	150 ± 2	6±1	18 ± 1

$R_h(20\,^{\circ}\text{C})/R_h(40\,^{\circ}\text{C}) = 0.966 \cdot f^{-0.2}$ [6] to estimate f.

For the ultra-low crosslinked microgels used here, we obtain $f = 0.35\,\%$. When this value is compared to the data reported in literature and collected in Ref. [6], we see that for regularly crosslinked microgels of comparable size to the one used here, synthesized with a wt% of crosslinker equal to 0.89 wt% [7], f results to be equal to 1.32 mol%. Also for the other microgels reported in Ref. [6], f always results in values larger than 0.35%. This confirms that the ULC microgels have the polymeric network that contains the lowest amount of crosslinker.

Supplementary Note IV: Comparison between hard and fuzzy sphere model above the VPTT.

We have chosen to use the fuzzy sphere model to fit the form factor above the VPTT to not impose any *a priori* knowledge to our data. Doing this, we let all the parameter of the model, particularly the length of the fuzzy shell, free to change. As expected, the external fuzziness disappears reaching a value of (2 ± 1) nm. To further support our results, Supplementary Figure 2 shows the comparison between the fits (a) and the resulting size distributions (b) obtained from fitting the SANS data (red points in (a)) to the fuzzy sphere and to the hard sphere model. As can be seen, both the data fits and the radial distribution of the relative polymer volume fraction within the microgels are virtually the same.

Supplementary Note V: Determination of the mesh size.

In regular microgels the addition of crosslinker agent leads to a more crosslinked core and a fuzzy shell with fewer crosslinks with respect to the core. ULC have a much lower content of cross-links as compared to microgels prepared with a crosslinking agent. Consequently, the fuzzy shell of regular microgels is more crosslinked compared to the ULC microgels despite the fact that the ULC microgels have an almost uniform polymer distribution surrounded by a small fuzzy shell.
To support this hypothesis, we can estimate the mesh size of the polymeric network of the microgels using the high-q region of the SANS scattered intensity for the 5 mol% crosslinked microgels used in the main manuscript, for microgels synthesized with 1 mol% BIS and for the ULC microgels. Supplementary Figure 3 shows the SANS intensities, $I(q)$, and the fit of the data with:

$$I(q) = \frac{I_L(0)}{1 + \xi^2 q^2}\,, \tag{1}$$

where $I_L(0)$ is intensity at $q = 0$ and ξ is the mesh size of the network [8, 9]. From the fits we obtain that the ULC microgels have $\xi_{ULC} = 24 \pm 1$ nm while for the 1 and 5 mol% crosslinked microgels we obtain $\xi_{1\ \text{mol\%}} = 7.4 \pm 0.4$ nm and $\xi_{5\ \text{mol\%}} = 6.7 \pm 0.3$ nm, respectively. Those values are average values for the mesh size between the core and the shell. Nevertheless they prove that, in average, ULC microgels presents a network with larger meshes. We would like to specify that as highlighted by SANS, ULC microgels posses a fuzzy external shell, as well. Both, the homogeneous region and the fuzzy shell of ULC microgels are expected to be less crosslinked than the corresponding regions in a regular crosslinked microgels. This is reflected in a larger average mesh size. Therefore, it is reasonable to assume that ULC microgels have a more penetrable polymeric network.

Supplementary Note VI: Viscosimetry.

The mass concentration of microgels in suspension, c, is linked to their generalized volume fraction, ζ, by a conversion constant, k: $\zeta = kc$. This constant is obtained by measuring the viscosity of suspensions of microgels at low concentrations, between 0.1 and 0.4 wt%.

To obtain the viscosity, the average time of fall, t, of a constant volume of microgel suspension through a thin capillary of an Ubbelohde tube viscometer immersed in a water bath at a fixed temperature of $(20.0 \pm 0.1)\,^{\circ}$C is measured. The average times of fall of the suspensions at different concentrations are linked to the kinematic viscosities, ν, by a constant, C, that only depends on the geometry of the capillary: $\nu = Ct$; in our experiments, $C = 3.156 \cdot 10^{-9}\,\text{m}^2\ \text{s}^{-2}$. By knowing the sample density, that can be approximated with the one of water

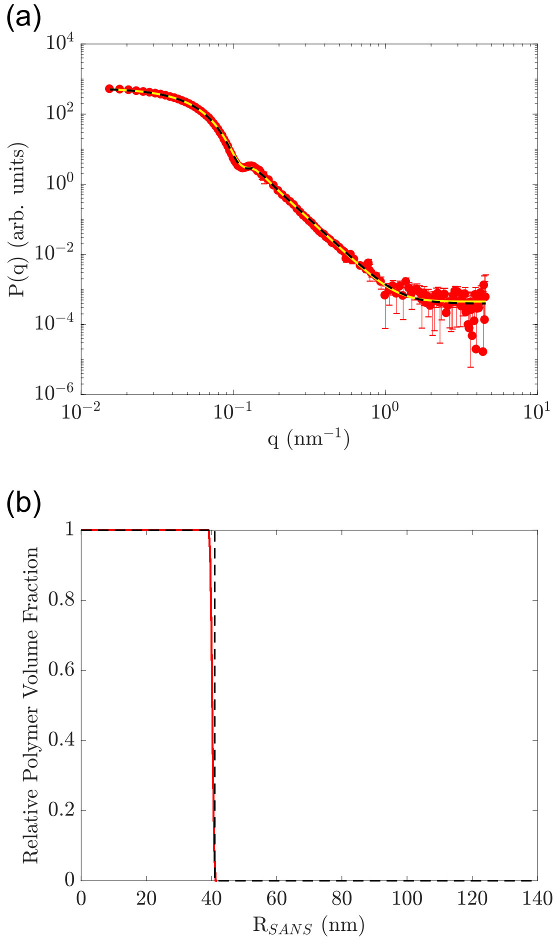

Supplementary Figure 2. SANS data and radial profiles. (a) Small-angle neutron scattering form factor, $P(q)$, versus scattering vector, q, for ultra-low crosslinked microgels in dilute suspensions of D_2O at $(40.0 \pm 0.5)\,^{\circ}$C, red squares. The solid lines are fits with the fuzzy sphere model (yellow) and with the hard sphere model (black dashed) [8]. (b) Relative polymer volume fraction versus radius obtained from the data fits for ultra-low crosslinked microgels at $(40.0 \pm 0.5)\,^{\circ}$C from the fuzzy sphere model (red solid line) and the hard sphere model (black dashed line).

(ρ_{H_2O}) due to the low concentration of microgels in all the measured suspensions, the viscosity of the microgel suspensions as a function of microgel concentration can be computed, $\eta = \nu \rho_{H_2O}$.

The experimental data for the relative viscosity, $\eta_r = \eta/\eta_{\mathrm{solvent}}$, ○, are shown in Supplementary Figure 4 for ULC (a) and regular 5 mol% crosslinked microgels (b). The solid lines represent fit with the Batchelor-Einstein equation: $\eta_r = 1 + 2.5\zeta + 5.9\zeta^2 = 1 + 2.5kc + 5.9(kc)^2$ [10]. The values of k for the different microgels at $(20.0 \pm 0.1)\,^{\circ}$C are obtained from the fits, and listed in the last column of Supplementary Table I.

Supplementary Note VII: Rheology.

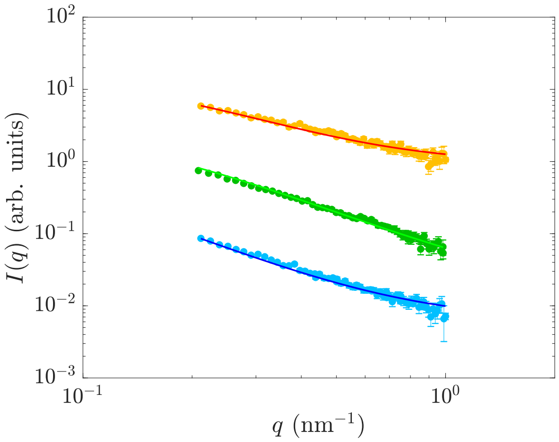

Supplementary Figure 3. High-q SANS data and fits. SANS intensities for 5 mol% crosslinked microgels used in the main manuscript (orange circles), for microgels synthesized with 1 mol% BIS (green circles) and for the ULC microgels (light blue). The solid line are fits of the data using Eq. 1

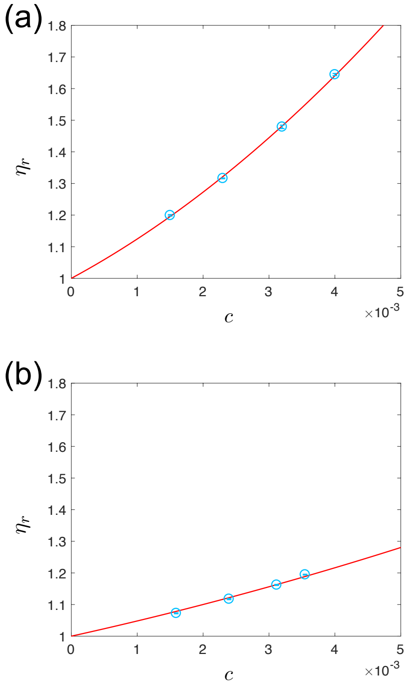

Supplementary Figure 4. Viscosimetry data and fits. Relative viscosity, η_r, versus the concentration of the microgels in suspension, c, (○) for: (a) ultra-low crosslinked microgels; (b) regular 5 mol% crosslinked microgels. The solid lines represent the fit of the data with the Batchelor-Einstein equation [10]

Oscillatory rheology has been used to probe the flow properties of the suspensions. Before each oscillatory experiments, test measurements were performed to verify that the suspensions were in the linear viscoelastic regime

at the frequencies $\omega = 0.1$, 1, and 10 rad s^{-1}. At the end of the oscillatory experiments a final test measurement at $\omega = 1$ rad s^{-1} was repeated to check that the system was still in the linear viscoelastic regime.

At $\zeta = 0.89 \pm 0.02$ the crossing point between the storage modulus, G' (■), and the loss modulus, G'' (○), is clearly visible in Supplementary Figure 5(a). For $\omega \lesssim 3$ rad s^{-1} the suspension behaves as a fluid with G'' (○) larger than G' (■). An increase of the frequency above 3 rad s^{-1} leads to $G' > G''$, as expected for a solid.

As shown in Supplementary Figure 5(b), with increasing the volume fraction up to $\zeta = 1.05 \pm 0.03$, G'' reaches the expected minimum while G' slightly increases with the frequency.

A further increase of ζ, up to 3.02±0.01, leads to higher values for both G' and G'' (Supplementary Figure 5(c)). The softness of the ULC microgels has no dramatic effects on the flow properties of the material.

A regular 5 mol% crosslinked microgel shows the same behavior but at a lower generalized volume fraction. Furthermore, the increase of the storage modulus for the regular 5 mol% crosslinked microgel is more abrupt with increasing the concentration of the suspension with respect to the ultra-low crosslinked microgels (Supplementary Figure 6). For $\zeta > 1.00 \pm 0.02$, the values of the plateau of G', G_p, have been plotted versus the generalized volume fraction Fig. 2(b) in the main article.

Supplementary Note VIII: Error propagation for the Langmuir-Blodgett measurements.

Two sources of errors are important in the Langmuir-Blodgett measurements: (i) the errors linked to the determination of the values of π and *Area*; (ii) the error related to the amount of microgels in the spreading solution and the amount that goes to the interface.

The nominal error related to a single measurement, as reported by the producer, is for the surface pressure, π, 0.1 μN and for the trough area 0.01 cm. Therefore, the error bars are within the symbols of the experimental points reported in Fig. 3 in the manuscript.

The shifts in x-axis mostly originate from the normalization of the trough area (*Area*) by the amount of microgels (*Mass*) added. The amount is calculated from the concentration and weight of the spreading solution. Differences of the spreading solution's concentration are a source of error to be considered. More important is a partial loss of microgels into the sub-phase during addition, which might occur as microgels are soluble in the sub-phase.

Deviations in the surface pressure π between measurements of the same system stem from miss placement of the probe (Wilhelmy plate), impurities in the Langmuir trough, contamination of the spreading solution and vibrations of the setup. Experiments were conducted with utmost care to eliminate these errors.

To evaluate the impact on those errors on the reproducibility of a measurements, we conducted multiple measurements (with and without simultaneous deposition) as you can see e.g. in Fig. 3 (cyan curves). From the repeated measurements, at least six independent measurements, we have computed the standard deviation for the surface pressure, σ_π, and for the *Area/mass*, σ_x. The obtained values are: $\sigma_\pi \lesssim 0.3$ mN m^{-1} and $\sigma_x \lesssim 1$ %. Supplementary Figure 7 shows the inset of Fig. 3 of the main manuscript showing the experimental errors. As can be seen the differences in the course of the curves are larger than the experimental errors. This confirms the robustness of our conclusions.

Supplementary Note IX: AFM and images analysis.

Obtained AFM images were processed with the analysis software *Gwyddion* 2.48 [11]. In Supplementary Figure 8 comparison between phase and height image are presented. Supplementary Figures 8(b) and (d) are the same height AFM micrographs as shown in Fig. 4(d)-(e). Supplementary Figures 8(a) and (c) are the corresponding phase images. In both, phase and height (Supplementary Figures 8(c) and (d)), the ULC microgels form uniform layers.

The roughness of the interface, γ_{RMS}, of ULC microgels was computed as the second moment of the height distribution, μ_2, over one AFM image using the free software *Gwyddion* according to the following equation [11]:

$$\gamma_{RMS} = \mu_2^{\frac{1}{2}} \tag{2}$$

The results are presented in Supplementary Figure 9. With increasing compression, increasing π, the microgels regain their shape and the roughness rises. In regime i the ULC microgels are separated and the roughness of the image is not computed. In regime ii, the microgels are virtually indistinguishable and we obtain $\gamma_{RMS} <$ 300 pm, the same as the substrate (Supplementary Figure 9). With increasing π the roughness slowly increases (Supplementary Figure 9 iii) as the microgels are laterally compressed. In regime iv, γ_{RMS} increases steeply from $\approx$ 500 pm to 1.3 nm at the highest investigated surface pressure (Fig. 3(i)). At this point ($\pi = 28$ mN m^{-1}), compression leads to desorption of polymer subchains from the interface.

The AFM micrographs, such as in Fig. 4, of ultra-low crosslinked and regular 5 mol% crosslinked microgels were further analyzed with a custom-written Matlab script. After locating the center of each microgel with the public available particle tracking code by Crocker and Grier [12], a Delaunay triangulation was performed. The 2D hexagonal order parameter, ψ_6, was calculated for

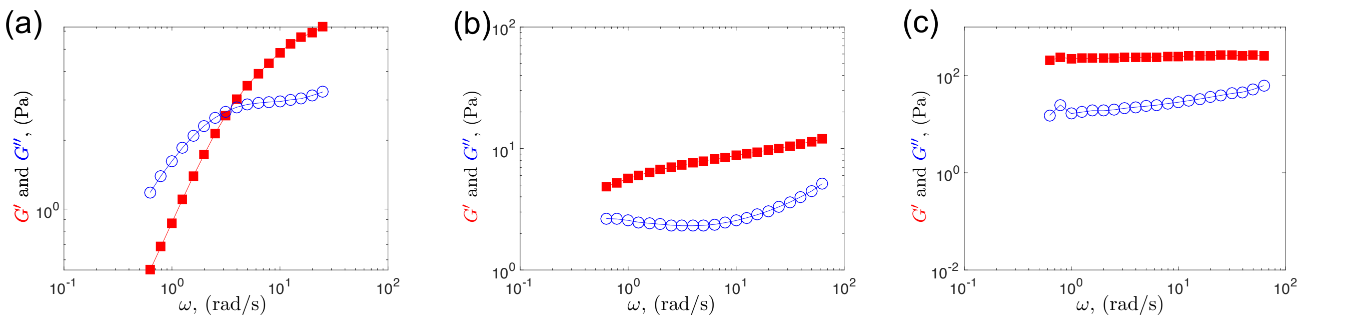

Supplementary Figure 5. Rheology measurements of ULC microgels. Storage (G', ■) and loss (G'', ○) moduli versus oscillation frequency, ω, for $\zeta = 0.89 \pm 0.02$ (a), $\zeta = 1.05 \pm 0.03$ (b), and $\zeta = 3.02 \pm 0.01$ (c).

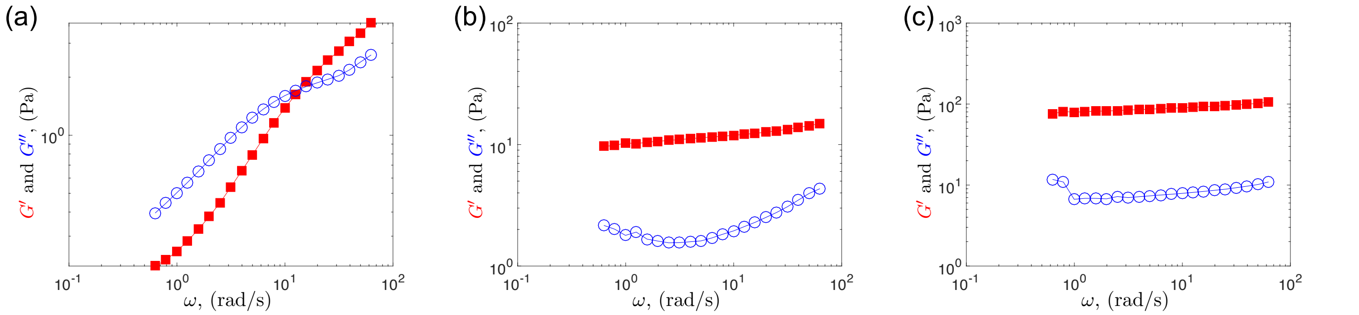

Supplementary Figure 6. Rheology measurements of regularly crosslinked microgels. Storage (G', ■) and loss (G'', ○) moduli versus oscillation frequency, ω, for regular 5 mol% crosslinked microgels at $\zeta = 0.60 \pm 0.02$ (a), $\zeta = 0.70 \pm 0.02$ (b), $\zeta = 0.80 \pm 0.02$ (c).

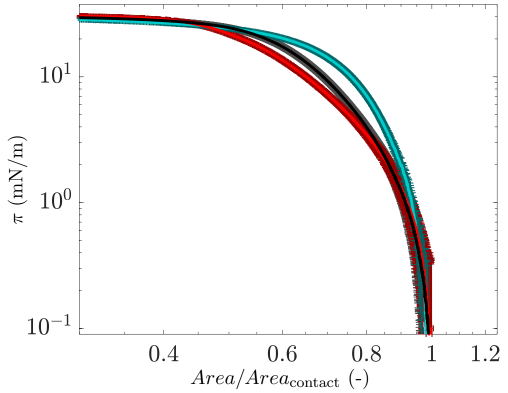

Supplementary Figure 7. Plot of π versus the trough area normalized by the trough area at the contact point, $Area/Area_{\text{contact}}$, of 5 mol% crosslinked microgels (cyan lines), ULC microgels (black lines) and linear pNIPAM (red lines) at $T = (20.0 \pm 0.5)$ °C in log-log scale including errors.

the microgels relative to a specific axis using the following formula:

$$\psi_6 = \left\langle \frac{1}{N_b} \left| \sum_{j=1}^{N_b} e^{(in\theta_j)} \right| \right\rangle, \quad (3)$$

where N_b is the number of nearest neighbors, n is set to 6 and θ_j is the phase angle between each microgel and its nearest neighbor j.

In Supplementary Figure 10 grayscale AFM images of the depositions at different compressions are presented. The markers give the positions of the determined centroids and the color the phase angle of the individual microgels with respect to their nearest neighbors. Supplementary Figure 10(a) shows the phase angle map of the ultra-low crosslinked microgels displayed in Fig. 4(f). Just by the naked eye a disordered lattices is visible, further underlined by the broad variation of the colors. In Supplementary Figure 10(b) the compressed ULC microgel monolayer (Fig. 4(i)) is presented, again a disordered structure can be seen. In Supplementary Figure 11 the order parameter is shown as a function of the *Area/Mass*, emphasizing the difference to regular crosslinked microgels [13].

For the regular 5 mol% crosslinked p(NIPAM) microgels phase angle maps at the contact and at high compression are given in Supplementary Figure 10(c) and (d), respectively. The image analysis for these depositions results in ψ_6 values for ordered (Fig. 4(b)) of 0.95 ± 0.02 and for disordered/polycrystalline lattices (Fig. 4(c)) of 0.58 ± 0.22. This transition is due to the formation of crystalline domains which then evolve in a more compact

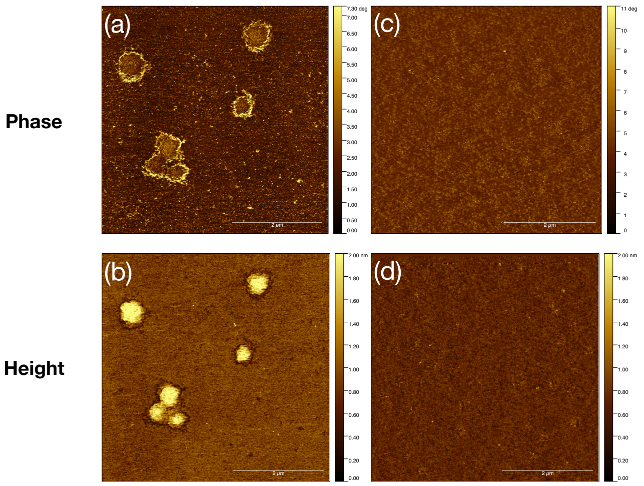

Supplementary Figure 8. AFM micrographs. AFM images of the ULC microgels before (a)-(b) and at contact (c)-(d). Phase images are in the upper, height images are in the lower row.

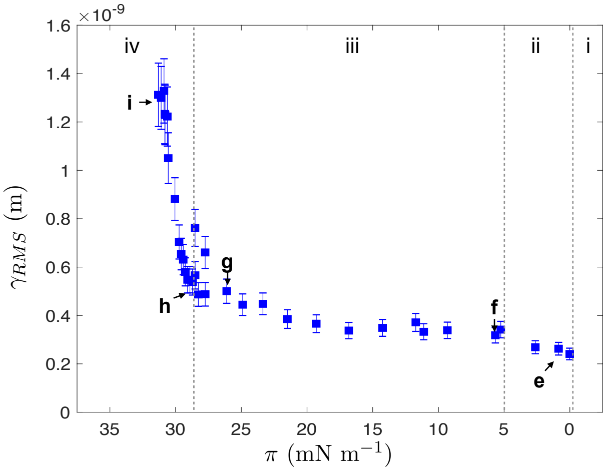

Supplementary Figure 9. Roughness γ_{RMS} of the deposited ultra-low crosslinked microgel monolayers in dry state as a function of surface pressure π. Positions of the images in Fig. 4(d)-(i) are highlighted. Gray dashed lines delimit the different visible regimes: (i) ULC microgels are not in contact, γ_{RMS} was not determined; (ii) ULC microgels are indistinguishable; (iii) steep increase in surface pressure, but only small increase in γ_{RMS}, as the ULC microgels are laterally compressed; (iv) γ_{RMS} increases steeply as compression in lateral dimensions becomes unfavorable and more and more subchains are pushed out of plane.

form as explained in Ref. [13] and shown in Supplementary Figure 13.

The reason for the suppression of crystallization in two-dimensions is due to the fact that once adsorbed to the interface, the ULC microgels show an heterogeneous stretching with a consequent increase in the polydispersity above the value that suppresses crystallization for hard disks in two-dimensions [14]. With increasing the compression of the monolayer of ULC microgels their size distribution becomes narrower, but still too broad to allow crystals to form. Even assuming that a higher compression further decreases the polydispersity, microgels are jammed together making any rearrangements impossible and the materials further fails to crystallize.

To support this, we have estimated the size distributions of ULC and regular 5 mol% crosslinked microgels at different degree of compressions (Supplementary Figure 12). Supplementary Figure 12(a) shows the radial distribution for the ultra-low crosslinked microgels when they are not in contact, e.g. Fig. 4(d). Several images have been recorded to improve the statistics. The overall result for 64 microgels leads to a size polydispersity of

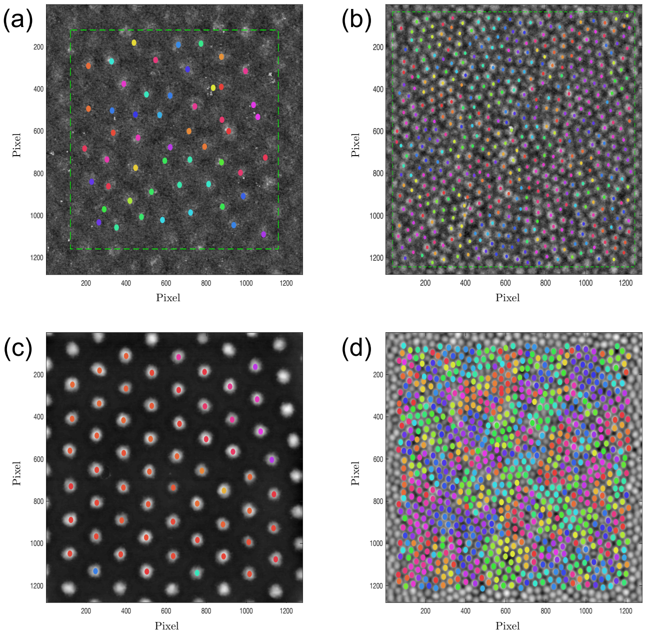

Supplementary Figure 10. Phase angle maps of ULC and regular 5 mol% crosslinked microgels. (a) ULC corresponding to Fig. 4(f); (b) ULC corresponding to Fig. 4(i); (c) regular 5 mol% crosslinked microgels corresponding to Fig. 4(b); (d) regular 5 mol% crosslinked microgels corresponding to Fig. 4(c). The markers show the positions of determined centroids and their color represent the local phase angle with the nearest neighbors. Single microgels were only accounted for if their centroids are inside the green dashed box as drawn in (a) and (b).

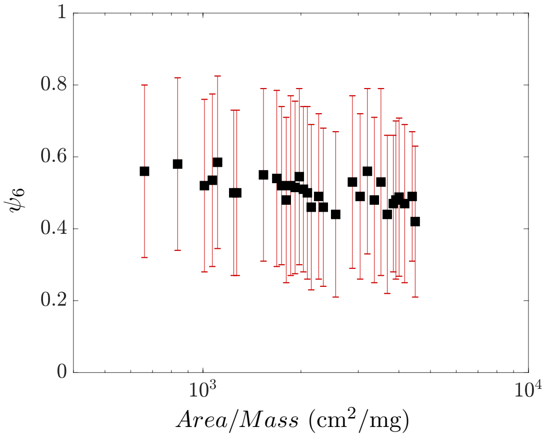

Supplementary Figure 11. The order parameter ψ_6 as a function of the *Area/Mass*.

(23 ± 5) %.

Under compression, the ULC microgels show a narrower size distribution. Supplementary Figure 12(b) shows the results for the analysis of the image in (Fig. 4(i)), with a size polydispersity of (18.0 ± 0.5) %. This is due to the fact that the softer microgels are compressed first with a consequent decrease of the size polydispersity, as in the three dimensional case [15, 16]. In contrast, the 5 mol% crosslinked microgels display narrow size distributions with polydispersity $\lesssim 11\,\%$, independently on the compression as shown in Supplementary Figure 12(c)-(d).

Supplementary Note X: Solid-solid transition of the monolayer of regular microgels.

An AFM micrograph taken in position (m) of the compression curve of the 5 mol% crosslinked microgels

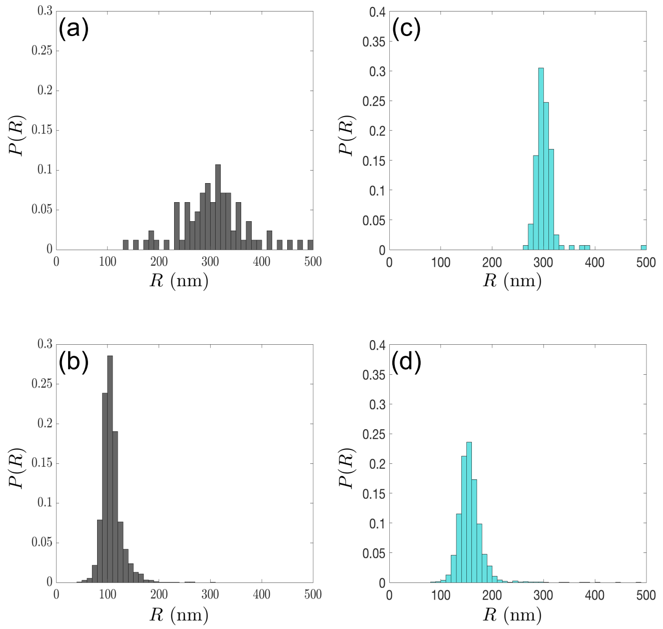

Supplementary Figure 12. Distributions *P(R)* versus *R(nm)* of ULC (a)-(b) and regular crosslinked microgels (c)-(d). In (a) *R(nm)* represents the interfacial radius measured from 64 ULC microgels. In (b)-(d) *R(nm)* represents the half of the nearest neighbor distances.

is reported in the top right of Supplementary Figure 13. From this image is clear that the previous crystalline structure, characterized by a larger lattice constant, is melting and at the same time crystalline grains of the new crystals are forming. These grains are characterized by a smaller lattice constant due to the compression of the fuzzy shell.

To further prove this, the radial distribution functions corresponding to the different crystalline phases, (b) and (c) are reported in the bottom line of Supplementary Figure 13. The two crystals are characterized by two different lattice constants, first peak in (b) and (c), corresponding to 590 and 150 nm. The dramatic decrease of the size is due to the compression of the microgels at the interface due to the movement of the barriers. To further highlight that, Supplementary Figure 13 reports the probability distribution for the nearest neighbor distance, $P(d_{nn})$, corresponding to the AFM micrograph at position (m). During the transition two distinct nearest neighbor distances emerge, one corresponding to the new lattice and one corresponding to the initial crystals that are melting. This is clearly demonstrated by the presence of a bimodal distribution in $P(d_{nn})$. The fist peak, d_{nn} = 180 nm, correspond to the new lattice while the second peak, d_{nn} = 350 nm, is related to the melting crystals.

The fact that we have two crystalline phases, characterized by two significantly different lattice constants and that the second lattice forms while the first one is melting, is consistent with studies of microgels at interfaces that observed the same behavior [13, 17, 18].

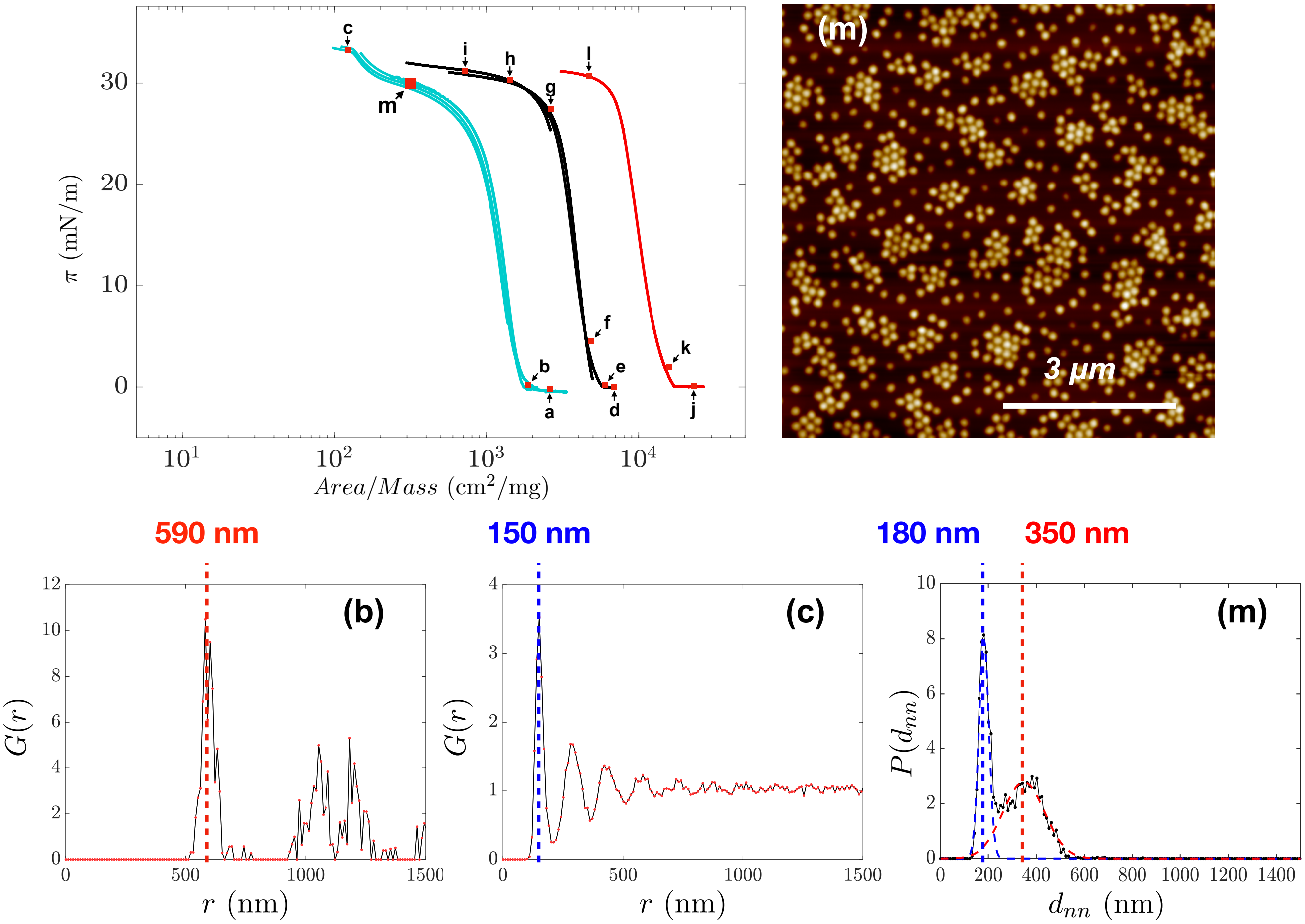

Supplementary Figure 13. Top left: compression isotherms as reported in Fig. 3 in the manuscript where it is marked the point corresponding to the AFM micrograph in (m), top right. Bottom: radial distribution functions corresponding to panels (b) and (c) of Fig. 4 in the main manuscript and probability distribution for nearest neighbor distance, d_{nn}, during the phase transition in image (m).

* richtering@rwth-aachen.de

Supplementary References

[1] D. E. Koppel. Analysis of macromolecular polydispersity in intensity correlation spectroscopy: The method of cumulants. *J. Chem. Phys.*, **57,** 4814–4820 (1972).

[2] W. Burchard & W. Richtering. *Dynamic light scattering from polymer solutions*, Steinkopff, Darmstadt (1989).

[3] S. Provencher. Inverse problems in polymer characterizaion: Direct analysis of polydispersity with photon correlation spectroscopy. *Makromol. Chem.*, **180,** 201–209 (1978).

[4] S. Provencher. A constrained regularization method for inverting data represented by linear algebraic or integral equations. *Computer Physics Communications*, **27,** 213–227 (1982).

[5] A. Scotti, W. Liu, J. S. Hyatt, E. S. Herman, H. S. Choi, J. W. Kim, L. A. Lyon, U. Gasser & A. Fernandez-Nieves. The CONTIN algorithm and its application to determine the size distribution of microgel suspensions. *J. Chem. Phys.*, **142,** 234905 (2015).

[6] C. G. Lopez & W. Richtering. Does Flory-Rehner theory quantitatively describe the swelling of thermoresponsive microgels? *Soft Matter*, **13,** 8271–8280 (2017).

[7] U. Gasser, J. S. Hyatt, J.-J. Lietor-Santos, E. S. Herman, L. A. Lyon & A. Fernandez-Nieves. Form factor of pnipam microgels in overpacked states *J. Chem. Phys.*, **141,** 034901 (2014).

[8] M. Stieger, W. Richtering, J. S. Pedersen, & P. Lindner. Small-angle neutron scattering study of structural changes in temperature sensitive microgel colloids. *J. Chem. Phys.*, **120,** 6197–6206 (2004).

[9] A. Fernandez-Barbero, A. Fernandez-Nieves, I. Grillo & E. Lopez-Cabarcos. Structural modifications in the swelling of inhomogeneous microgels by light and neutron scattering. *Phys. Rev. E*, **66,** 051803 (2002).

[10] G. K. Batchelor. The effect of Brownian motion on the bulk stress in a suspension of spherical particles. *J. Fluid. Mech.*, **83,** 97–117 (1977).

[11] P. Klapetek, D. Necas, & C. Anderson. *Gwyddion user guide* (2018). URL http://gwyddion.net/documentation/user-guide-en/.

[12] J. C. Crocker & D. G. Grier. Methods of Digital Video Microscopy for Colloidal Studies. *J. Colloid Interface Sci.*, **179,** 298–310 (1996).

[13] M. Rey, M. A. Fernandez-Rodriguez, M. Steinacher, L. Scheidegger, K. Geisel, W. Richtering, T. M. Squires & L. Isa. Isostructural solid-solid phase transition in monolayers of soft core-shell particles at fluid interfaces: structure and mechanics. *Soft Matter* **12,** 3545–3557 (2016).

[14] S. Pronk & D. Frenkel Melting of polydisperse hard disks *Phys. Rev. E.*, **69,** 066123 (2004).

[15] A. Scotti, U. Gasser, E. S. Herman, M. Pelaez-Fernandez, L. A. Lyon & A. Fernandez-Nieves. The role of ions in the self-healing behavior of soft particle suspensions. *PNAS* **113,** 5576-5581 (2016).

[16] A. Scotti, U. Gasser,E. S. Herman, J. Han, A. Menzel, L. A. Lyon & A. Fernandez-Nieves. Premelting at Defects Within Bulk Colloidal Crystals. *Phys. Rev. E* **96,** 032609 (2017).

[17] C. Picard, P. Garrigue, M.-C. Tatry, V. Lapeyre, S. Ravaine, V. Schmitt & V. Ravaine. Organization of Microgels at the Air-Water Interface under Compression: Role of Electrostatics and Cross-Linking Density. *Langmuir*, **33,** 7968–7981 (2017).

[18] L. Scheidegger, M. A. Fernandez-Rodriguez, K. Geisel, M. Zanini, R. Elnathan, W. Richtering & L. Isa. Compression and deposition of microgel monolayers from fluid interfaces: particle size effects on interface microstructure and nanolithography. *Phys. Chem. Chem. Phys.*, **19,** 8671 (2017).
